# Clinical and molecular factors that impact the efficacy of first-line crizotinib in ROS1-rearranged non-small-cell lung cancer: a large multicenter retrospective study

**DOI:** 10.1186/s12916-021-02082-6

**Published:** 2021-09-13

**Authors:** Yongchang Zhang, Xiangyu Zhang, Ruiguang Zhang, Qinqin Xu, Haiyan Yang, Analyn Lizaso, Chunwei Xu, Jun Liu, Wenxian Wang, Sai-Hong Ignatius Ou, Jiexia Zhang, Zhengbo Song, Nong Yang

**Affiliations:** 1 Department of Medical Oncology, Lung Cancer and Gastrointestinal Unit, Hunan Cancer Hospital/The Affiliated Cancer Hospital of Xiangya School of Medicine, Central South University, Changsha, 410013 China; 2 Graduate School, University of South China, Hengyang, 421001 Hunan China; 3 Cancer Center, Union Hospital, Tongji Medical College, Huazhong University of Science and Technology, Wuhan, 430022 China; 4 Department of Medical Oncology, Qinghai Provincial People’s Hospital, Xining, 810000 China; 5 Burning Rock Biotech, Guangzhou, 510300 China; 6 Department of Respiratory Medicine, Jinling Hospital, Nanjing University School of Medicine, Nanjing, China; 7 Department of Medical Oncology, Cancer Hospital of the University of Chinese Academy of Sciences (Zhejiang Cancer Hospital), Zhejiang, 310022 Hangzhou China; 8 Chao Family Comprehensive Cancer Center, Department of Medicine, Division of Hematology-Oncology, University of California Irvine School of Medicine, Orange, CA USA; 9 National Clinical Research Center for Respiratory Disease, State Key Laboratory of Respiratory Disease, Department of Medicine, Guangzhou Institute of Respiratory Disease, Guangzhou Institute of Respiratory Health, Guangzhou, 510120 China

**Keywords:** ROS1 gene rearrangements, Crizotinib, Concomitant mutations, Progression associated efficacy

## Abstract

**Background:**

*ROS1*-rearranged lung cancers benefit from first-line crizotinib therapy; however, clinical and molecular factors that could affect crizotinib efficacy in *ROS1*-rearranged lung cancers are not yet well-elucidated. Our retrospective study aimed to compare the efficacy of chemotherapy and crizotinib in the first-line treatment of *ROS1*-rearranged advanced lung cancer and evaluate various clinical and molecular factors that might impact crizotinib efficacy in real-world practice.

**Methods:**

Treatment responses, survival outcomes, and patterns of disease progression were analyzed for 235 patients with locally advanced to advanced disease who received first-line chemotherapy (*n* = 67) or crizotinib (*n* = 168).

**Results:**

The overall response rate was 85.7% (144/168) for first-line crizotinib and 41.8% (28/67) for chemotherapy. Patients treated with first-line crizotinib (*n* = 168) had significantly longer median progression-free survival (PFS) than chemotherapy (*n* = 67) (18.0 months vs. 7.0 months, *p* < 0.001). Patients harboring single *CD74-ROS1* (*n* = 90) had significantly shorter median PFS with crizotinib than those harboring non-*CD74 ROS1* fusions (*n* = 69) (17.0 months vs. 21.0 months; *p* = 0.008). Patients with baseline brain metastasis (*n* = 45) had a significantly shorter PFS on first-line crizotinib than those without brain metastasis (*n* = 123) (16.0 months vs. 22.0 months; *p* = 0.03). At progression, intracranial-only progression (*n* = 40), with or without baseline CNS metastasis, was associated with longer median PFS than those with extracranial-only progression (*n* = 64) (19.0 months vs. 13.0 months, *p* < 0.001). *TP53* mutations were the most common concomitant mutation, detected in 13.1% (7/54) of patients with *CD74-ROS1* fusions, and 18.8% (6/32) with non-*CD74 ROS1* fusions*.* Patients with concomitant *TP53* mutations (n=13) had significantly shorter PFS than those who had wild-type *TP53* (*n* = 81) (6.5 months vs. 21.0 months; *p* < 0.001). PFS was significantly shorter for the patients who harbored concomitant driver mutations (*n* = 9) (11.0 months vs 24.0 months; *p* = 0.0167) or concomitant tumor suppressor genes (i.e., *TP53*, *RB1*, or *PTEN*) (*n* = 25) (9.5 months vs 24.0 months; *p* < 0.001) as compared to patients without concomitant mutations (*n* = 58).

**Conclusion:**

Our results demonstrate that baseline brain metastatic status and various molecular factors could contribute to distinct clinical outcomes from first-line crizotinib therapy of patients with *ROS1*-rearranged lung cancer.

**Clinical trials registration:**

CORE, NCT03646994

**Supplementary Information:**

The online version contains supplementary material available at 10.1186/s12916-021-02082-6.

## Background

Genomic rearrangements involving ROS proto-oncogene-1 (ROS1) are actionable targets in the treatment of non-small-cell lung cancer (NSCLC) [1]. The overall prevalence of *ROS1* fusions is reported to be 2% in NSCLC and up to 3.3% in lung adenocarcinoma [1–4]. *ROS1*, located at the long arm of chromosome 6q22, encodes one of the receptor tyrosine kinases of the insulin receptor family; however, its exact activating ligand remains unidentified and hence is considered an orphan receptor [4, 5]. Since the amino acid sequence of the kinase domains of ROS1 and ALK are highly homologous, selective inhibitors of ALK, including crizotinib, have shown anti-tumor activity in vitro and have been explored clinically in the treatment of patients with *ROS1*-rearranged tumors [5–13]. Crizotinib has been approved for use in *ROS1*-rearranged NSCLC based on the PROFILE 1001 study, which observed an objective response rate (ORR) of 72%, median progression-free survival (PFS) of 19.2 months, and median overall survival of 51.4 months among the 53 patients with *ROS1*-rearranged NSCLC included in the expansion cohort [8, 14]. Meanwhile, the largest phase II study conducted to date on crizotinib response of *ROS1*-positive NSCLC demonstrated an ORR of 71.7% with a median PFS of 15.9 months among 127 East Asian patients [13]. Given the rarity of *ROS1* fusions, the effect of *ROS1* fusion variants and other clinical and molecular factors on the efficacy of crizotinib is not well-elucidated. So far, the real-world studies describing the efficacy of crizotinib in Asian patients with *ROS1*-rearranged NSCLC mostly involved smaller cohorts.

This retrospective cohort study aimed to explore the efficacy of crizotinib as a first-line treatment for advanced NSCLC with various *ROS1* rearrangements. We also investigated clinical and molecular factors that could impact the clinical outcomes of patients with locally advanced to advanced *ROS1*-rearranged NSCLC from first-line crizotinib therapy.

## Patients

### Included patients

We retrospectively screened 21,747 consecutive treatment-naïve patients who were diagnosed with lung cancer from August 1, 2018, to March 31, 2020, and submitted samples for molecular detection of *ROS1* rearrangements, including next-generation sequencing (NGS), fluorescence in situ hybridization (FISH), and amplification refractory mutation system (ARMS) at various hospitals in Hunan, Hubei, Guangdong, and Zhejiang Provinces in China. All the patients who were analyzed for clinical outcomes from first-line crizotinib met the following criteria: (1) pathologically-confirmed NSCLC, (2) locally advanced/unresectable disease to advanced/metastatic disease; (3) *ROS1* rearrangements identified by NGS, and (4) treatment with crizotinib in the first-line setting. Written informed consent was obtained from all the patients for the use of their data for research purposes. All procedures in our study were performed following the ethical standards of the institutional and national research committees, and the Declaration of Helsinki as revised in 2013. Approval was obtained from the Hunan Cancer Hospital Institutional Review Board Committee (approval number: 2017YYQ-SSB-026). This study was also registered as a clinical trial (CORE, NCT03646994).

### NGS

Patient samples were submitted for NGS-based analysis to Burning Rock Biotech, a College of American Pathologists-accredited, Clinical Laboratory Improvement Amendments -certified clinical laboratory. Briefly, DNA isolated from the tissue biopsy, blood, or pleural effusion samples obtained from all the patients were processed accordingly for NGS using commercially available panels targeting various cancer-related genes and sequenced on a Nextseq 500 (Illumina, CA, USA) with paired-end reads with a target sequencing depth of 1000X for tissue samples and 10,000X for liquid biopsy samples using optimized protocols [15]. All the gene capture panels used in our study, including 8, 56, 108, 168, and 295 cancer-related genes, interrogated whole exons and critical introns for the 8 classic NSCLC oncogenic drivers, which includes *EGFR*, *ALK*, *BRAF*, *ERBB2*, *KRAS*, *MET*, *RET*, and *ROS1*. To understand the impact of concomitant mutations, including *TP53*, other tumor suppressor genes, and oncogenic driver genes, on crizotinib outcomes, we selected the patient samples that were assayed using 168-gene or 295-gene panels (*n* = 94). We only analyzed the 168 genes common between the 2 panels. Table S1 lists the genes included in the 168 gene panel. The sequencing analyses were performed using optimized bioinformatics pipelines for somatic variant calling that involved accurate identification of base substitutions, small insertions-deletions, copy number variations, and genomic rearrangements as described previously [15].

Non-reciprocal/reciprocal translocations are defined by the simultaneous detection of at least one *ROS1* fusion that contains the tyrosine kinase domain and another *ROS1* fusion involving the 5′-region of *ROS1* fused with other gene partners aside from the gene partner fused to the ROS1 tyrosine kinase domain, as previously described with *ALK* fusions [16].

### Evaluation of treatment efficacy

Crizotinib was orally administered at a dose of 250 mg twice daily. Chemotherapy with either pemetrexed-carboplatin regimen or docetaxel-cisplatin regimen was administered intravenously. The dose for pemetrexed was 500 mg per square meter of body-surface area (m^2^), plus carboplatin at a dose of target area under the curve of 5 to 6 mg per milliliter per minute. The dose for docetaxel was 75 mg/m^2^ and 75 mg/m^2^ for cisplatin. The treatment regimens were administered until progressive disease (PD) or unacceptable toxicity. Patients who experienced toxicity were managed by dose reduction or discontinuation as decided by their physicians. The best responses were assessed by the investigators according to the Response Evaluation Criteria in Solid Tumors version 1.1. For patients treated with crizotinib, as local law for drug purchase, all patients were required to undergo radiologic imaging evaluation including chest computed tomography (CT) scanning and brain magnetic resonance imaging (MRI) every 2 cycles. For patients treated with chemotherapy, as they were all treated in hospital, all patients were required to undergo chest radiologic imaging evaluation including chest CT and brain MRI every 2 cycles. The overall response rate (ORR) was calculated as the proportion of patients with complete response (CR) or partial response (PR). The disease control rate (DCR) was calculated as the proportion of patients with CR, PR, and stable disease (SD). Progression-free survival (PFS) was measured from the start of crizotinib administration until the date of PD or death from any causes. Each image was independently evaluated by two radiologists. The data cutoff date was December 31, 2020. The median follow-up duration was 28 (range: 2–59) months.

### Statistical analysis

Continuous variables were summarized as means and standard deviations or medians with range and compared using unpaired *t* test or Wilcoxon signed-rank test. Categorical variables were summarized as frequencies with percentages and compared using Chi-squared or Fisher’s exact test, as appropriate. Kaplan-Meier analysis was used to estimate the survival functions and log-rank test to determine the difference in survival outcomes between groups. The Cox proportional hazards model was used for multivariate survival analysis. Variables with a *p* value < 0.2 in the univariate analysis were included in the multivariate analysis. Schoenfeld residuals were used to check the proportional hazards assumption. All tests were two-sided, and *p* value < 0.05 was considered statistically significant. All statistical analyses were performed with R (version 3.3.3, the R Foundation for Statistical Computing, Vienna, Austria) and RStudio (version 1.1.383).

## Results

### Patient characteristics and distribution of ROS1 fusions identified through NGS

**Fig. 1:**
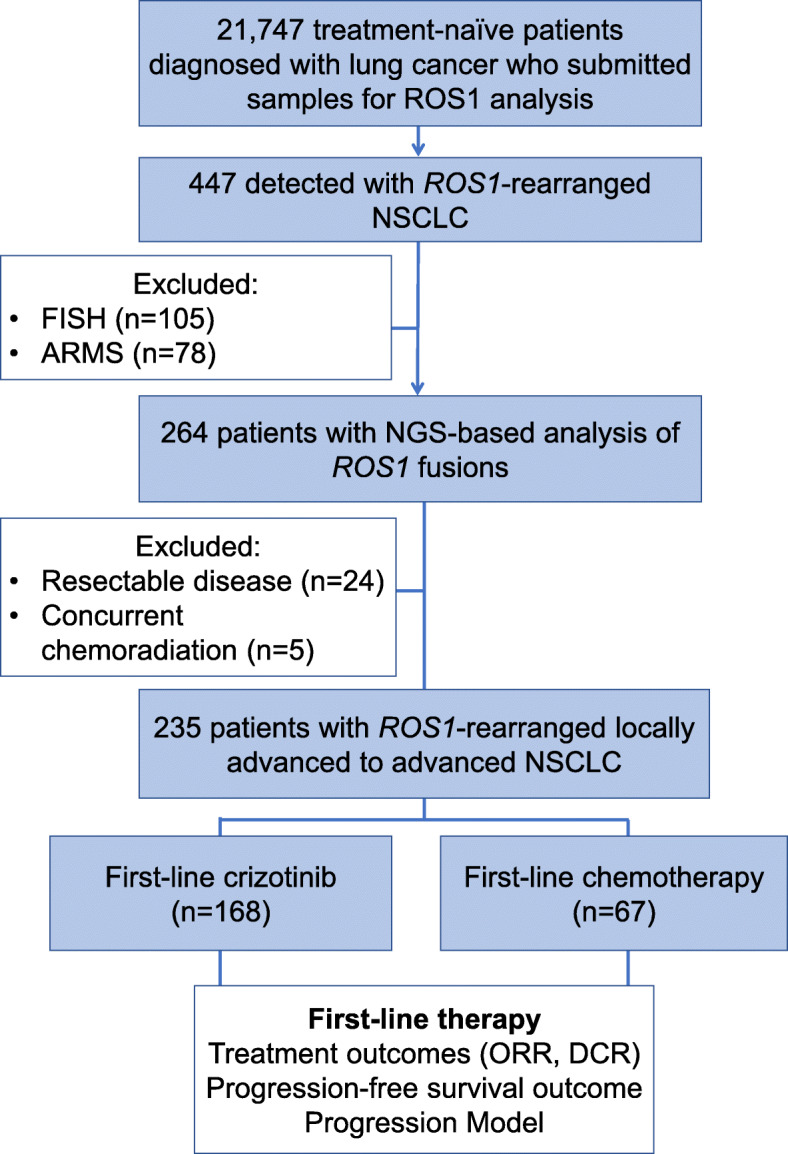
Study design schematic. Flow chart illustrating the study design. A total of 21,747 treatment-naïve patients diagnosed with lung cancer were retrospectively screened for *ROS1* fusion variants. Among them, 447 were detected with *ROS1* rearrangements using various methods including NGS (*n* = 264), FISH (*n* = 105), and ARMS (*n* = 78). Clinical outcomes were further analyzed for the 235 patients with *ROS1*-rearranged locally advanced to advanced lung cancer who received either chemotherapy (*n* = 67) or crizotinib (*n* = 168) as first-line therapy

Figure 1 summarizes our study design. Among the 21,747 consecutive treatment-naïve patients diagnosed with various stages of lung cancer who submitted samples for *ROS1* detection, 447 were positive for *ROS1* rearrangements, revealing an overall prevalence rate of 2.1%. A majority of the *ROS1* rearrangements were identified using NGS (59.1%; *n* = 264), while 23.5% (*n* = 105) were identified by FISH, and 17.4% (n=78) were identified by ARMS. Of the 264 patients who were identified as *ROS1* positive using NGS analysis, a majority (95.5%; *n* = 252) submitted tissue biopsy samples. The remaining 2.7% (*n* = 7) of the patients submitted plasma samples, and 1.8% (*n* = 5) submitted pleural effusion samples. Unfortunately, no patient submitted samples for all three molecular assays or multiple sample types to enable further concordance analyses among the detection methods or sample types.

Among the 235 patients with stage IIIB-IV disease, a majority was female (63%; *n* = 147), with a median age of 52 years (range: 25–79 years). Except for 1 patient having squamous cell carcinoma, all had adenocarcinoma (99.0%; *n* = 234). Of these 235 patients, 168 received crizotinib and 67 received platinum-based doublet chemotherapy regimen as first-line therapy. Baseline clinical characteristics including local treatment strategies were statistically similar among the subgroups (Table 1).

**Table 1:** Baseline clinicopathological characteristics of patients harboring *ROS1* fusions

	All *ROS1* fusion lung cancer patients (*n* = 447, *n*, %)	NGS identified ROS1 fusion lung cancer patients (*n* = 264, *n*, %)	Locally-advanced/advanced *ROS1* fusion lung cancer patients (*n* = 235, *n*, %)	Crizotinib, *n* = 168							Chemotherapy, *n* = 67							*p*
				All (*n* = 168, *n*, %)	Single *ROS1* fusion (*n* = 159)				Non-reciprocal/reciprocal *ROS1* translocation (*n* = 9)	*p*	All (*n* = 67, *n*, %)	Single *ROS1* fusion (n=61)				Non-reciprocal/reciprocal *ROS1* translocation (*n* = 6)	*p*	
					All (*n* = 159, *n*, %)	*CD74-ROS1* (*n* = 90, *n*, %)	Non-*CD74-ROS1* (*n* = 69, *n*, %)	*p*				All (*n* = 61, *n*, %)	*CD74-ROS1* (*n* = 40, *n*, %)	Non-*CD74-ROS1* (*n* = 21, *n*, %)	*p*			
Age (Median range)	52 (25–79)	52 (25–79)	52 (25–79)	52 (27–79)	52 (27–79)	52 (27–79)	52 (29–79)	0.868	54 (37–74)	0.793	52 (27–79)	53 (29–64)	54 (26–72)	54 (29–79)	0.837	54 (42–58)	0.889	0.837
Sex								0.443		0.487							0.662	0.508
Female	293 (66)	166 (63)	147 (63)	63(38)	61(38)	38 (42)	23 (33)		2 (2)		25 (37)	22 (36)	17 (43)	5 (24)	0.331	3 (50)		
Male	154 (34)	98 (37)	88 (37)	105(62)	98(62)	52 (58)	46 (67)		7 (98)		42 (63)	39 (64)	23 (57)	16 (76)		3 (50)		
Clinical stage								0.474		0.535					0.215		0.395	0.519
III	30 (7)	23 (9)	14 (6)	10(6)	9(6)	4 (4)	5 (7)		1 (1)		4 (6)	3 (5)	3 (8)	0 (0)		1 (16.7)		
IIIa	0 (0)	0 (0)	0 (0)	0(0)	0(0)	0 (0)	0 (0)		0 (0)		0 (0)	0 (0)	0 (0)	0 (0)		0 (0)		
IIIb	9 (2)	5 (2)	2 (1)	2(1)	1(1)	1 (1)	0 (0)		0 (0)		0 (0)	0 (0)	0 (0)	0 (0)		0 (0)		
IIIc	21 (5)	18 (7)	12 (5)	8(5)	8(5)	3 (3)	5 (7)		1 (1)		4 (6)	3 (5)	3 (8)	0 (0)		1 (16.7)		
IV	417 (93)	241 (91)	221 (94)	158(94)	150(94)	86 (96)	64 (93)		8 (99)		63 (94)	58 (95)	37 (92)	21 (100)		5 (83.3)		
Smoking status								0.284		0.487					0.33		0.933	
Smoker or former smoker	89 (20)	62 (23)	54 (23)	32(19)	29(18)	19 (21)	10 (14)		2 (2)		8 (12)	6 (10)	5 (13)	1 (5)		0 (0)		
Nonsmoker	358 (80)	202 (77)	181 (77)	136(81)	130(82)	71 (79)	59 (86)		7 (98)		59 (88)	55 (90)	35 (87)	20 (95)		6 (100)		
ECOG PS score								0.943		0.938					0.893		0.933	0.697
0–1	430 (96)	253 (96)	226 (96)	162(96)	153(96)	86 (96)	67 (97)		9 (100)		64 (96)	58 (95)	38 (95)	20 (95)		6 (100)		
≥ 2	17 (4)	11 (4)	9 (4)	6(4)	6(4)	4 (4)	2 (3)		0 (0)		3 (4)	3 (5)	2 (5)	1 (5)		0 (0)		
Tumor histology								0.998		0.974					/		/	
Adenocarcinoma	445 (99)	262 (99)	234 (99)	166(99)	157(99)	89 (99)	68 (99)		9 (100)		67 (100)	61 (100)	40(100)	21 (100)		6(100)		
Squamous cell carcinoma	2 (1)	2 (1)	1 (1)	2 (1)	2 (1)	1 (1)	1 (1)		0 (0)		0 (0)	0 (0)	0 (0)	0 (0)		0 (0)		
Presence of brain metastasis at baseline								0.83		0.735					0.928		0.666	0.747
Present	129 (29)	69 (26)	58 (25)	45 (27)	42 (26)	23 (26)	19 (28)		3 (33)		16 (24)	14 (23)	9 (23)	5 (24)		2 (33.3)		
Absent	318 (71)	195 (74)	177 (75)	123 (73)	117 (74)	67 (74)	53 (72)		6 (67)		51 (76)	47 (77)	31 (77)	16 (76)		4 (66.7)		
Method used for evaluating brain metastasis								0.129		0.474					0.924		0.845	0.796
CT	169 (38)	98 (37)	86 (37)	64 (38)	62 (39)	42 (47)	20 (29)		2 (22)		26 (39)	24 (39)	16 (40)	8 (38)		2 (33.3)		
MRI	278 (62)	166 (63)	149 (63)	104 (62)	97 (61)	48 (53)	49 (71)		7 (78)		41 (61)	37 (61)	24 (60)	13 (62)		4 (66.7)		
Local therapy received for management of brain metastasis								0.938		0.949					0.784		0.833	0.603
None	89 (69)	50 (72)	44 (76)	31 (69)	29 (69)	15 (65)	14 (74)		2 (67)		11 (69)	10 (71)	7 (78)	3 (60)		5 (83.3)		
WBRT	22 (17)	10 (14)	8 (14)	8 (18)	7 (17)	5 (22)	2 (11)		1 (33)		2 (13)	1 (7)	1 (11)	0 (0)		1 (16.7)		
SBRT	18 (14)	9 (14)	6 (10)	6 (13)	6 (14)	3 (13)	3 (15)		0 (0)		3 (18)	3 (22)	1 (11)	2 (40)		0 (0)		
Disease status								0.974		0.176					0.215		0.296	0.387
Non-progression	232 (52)	146 (55)	124 (53)	57 (34)	51 (32)	29 (32)	22 (32)		6 (67)		4 (6)	4 (7)	3 (8)	1 (5)		0 (0)		
Progression	215 (48)	118 (45)	111 (47)	111 (66)	108 (68)	61 (68)	47 (68)		3 (33)		63 (94)	57 (93)	37 (92)	20 (95)		6 (100)		
Site of progression								0.679		0.819					0.949		0.835	0.849
Brain	93 (43)	53 (45)	48 (43)	48 (43)	47 (44)	25 (41)	22 (47)		1 (33)		19 (30)	17 (30)	11 (30)	6 (30)		2 (33.3)		
Non-brain	122 (57)	65 (55)	63 (57)	63 (57)	61 (56)	36 (59)	25 (53)		2 (67)		44 (70)	40 (70)	26 (70)	14 (70)		4 (66.7)		

**Fig. 2:**
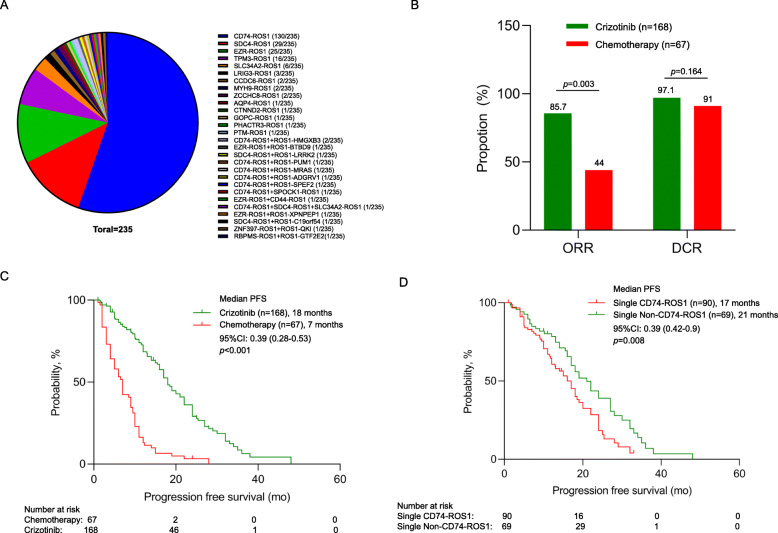
*ROS1*-rearranged NSCLCs had better objective response rate (ORR), disease control rate (DCR), and progression-free survival (PFS) with first-line crizotinib therapy than chemotherapy. **A** Distribution of various *ROS1* fusions detected using NGS-based method in 235 patients with locally advanced to advanced disease who received first-line therapy. **B** Treatment outcomes of patients with *ROS1*-rearranged NSCLCs who received either chemotherapy (red) or crizotinib therapy (green) in the first-line setting. **C**, **D** Kaplan-Meier survival curves illustrating the significantly better PFS for patients with *ROS1*-rearranged NSCLC (**D**) who received crizotinib (green) as compared to those who received chemotherapy (red) in the first-line setting; and (**E**) who received crizotinib and had non-*CD74 ROS1* fusions (green) as compared to those with single *CD74-ROS1* (red). The risk table below summarizes the number of patients included per time point

We further explored the various *ROS1* fusion variants and gene partners among the 235 patients with NGS data who received first-line therapy. A majority of the patients (93.6%, 220/235) were detected with only a single *ROS1* fusion partner, with *CD74-ROS1* as the most common (59.1%, 130/220), followed by *SDC4-ROS1* (13.2%, 29/220), and *EZR-ROS1* (11.4%, 25/220) (Table S2, Fig. 2A). Five previously unreported *ROS1* fusion partners were identified from our cohort, including *MYH9-ROS1* (*n* = 2), *AQP4-ROS1* (*n* = 1), *CTNND2-ROS1* (*n* = 1), *PHACTR3-ROS1* (*n* = 1), and *PTM-ROS1* (*n* = 1). Six percent (6.4%, 15/235) of the patients with *ROS1*-rearranged NSCLCs were detected with more than 1 genomic rearrangement involving *ROS1*, of which at least 1 of the rearrangements retained the kinase domain of ROS1, while the other *ROS1* fusion did not retain the kinase domain, which was referred to as non-reciprocal/reciprocal translocation. Among them, a majority had *CD74-ROS1* (47.1%; *n* = 8) as the *ROS1* fusion which retained the kinase domain. Interestingly, 1 patient was detected with 3 fusions (*CD74-ROS1*, *SDC4-ROS1*, and *SLC34A2-ROS1*), which all retained the kinase domain. Table S3 summarizes the detailed breakpoint information for the non-reciprocal/reciprocal *ROS1* translocations detected from our cohort**.**

### Comparison between chemotherapy and crizotinib as first-line therapy

Clinical outcomes were analyzed for the 235 patients with advanced or locally advanced *ROS1*-rearranged NSCLCs who received first-line therapy of either chemotherapy (*n* = 67; pemetrexed and carboplatin regimen (97%; *n* = 65), docetaxel and cisplatin regimen (*n* = 2)) or crizotinib (*n* = 168). As compared to patients who received chemotherapy (*n* = 67), patients who received crizotinib (*n* = 168) had significantly better ORR (85.7% (*n* = 144) vs 44% (*n* = 28), *p* = 0.003; Fig. 2B) and significantly longer median PFS (18.0 months vs. 7.0 months, *p* < 0.001, Fig. 2C).

### Impact of ROS1 fusion types on crizotinib efficacy

Next, we explored the clinical impact of *ROS1* fusion variants among the 168 patients with NGS data who received first-line crizotinib. Baseline clinicopathologic characteristics were similar for the patients regardless of *ROS1* fusion (Table 1).

PFS was significantly shorter for patients with single *CD74-ROS1* fusion (*n* = 90) than those with single non-*CD74 ROS1* fusions (*n* = 69) (17.0 months vs 21.0 months; *p* = 0.008; Fig. 2D). PFS was comparable among patients with single *CD74-ROS1* fusions (*n* = 90) and those with single *SDC4-ROS1* (*n* = 23; 16.0 months [95% confidence intervals (CI): 14.2–17.7 months]; *p* = 0.175), or single *EZR-ROS1* (n=21; 19.0 months [95% CI: 12.7–25.2 months]; *p* = 0.07), but was significantly longer for patients with uncommon non-*CD74 ROS1* fusions (*n* = 25; 22.0 months [95% CI: 14.5–29.5 months]; *p* = 0.02) (Figure S1A). The ORR (88.9% vs 82.6%; *p* = 0.92) and DCR (96.7% vs 97.1%; *p* = 0.96) was similar for patients with *CD74-ROS1* and non-*CD74 ROS1* fusions (Table S4). With first-line crizotinib therapy, the ORR was 91.3% for patients with *SDC4-ROS1* (*n* = 23), 85.7% for *EZR-ROS1* (*n* = 21), 91.7% for *TPM3-ROS1* (*n* = 12), and 88% for other uncommon non-*CD74 ROS1*, including *SLC34A2-ROS1* (*n* = 3), *LRIG3-ROS1* (*n* = 3), *MYH9-ROS1* (*n* = 1), *CCDC6-ROS1* (*n* = 1), *AQP4-ROS1* (*n* = 1), *CTNND2-ROS1* (*n* = 1), *PHACTR3-ROS1* (*n* = 1), *GOPC-ROS1* (*n* = 1), and *PTM-ROS1* (*n* = 1), which were grouped together due to small numbers. As compared to patients with single *ROS1* fusion (both *CD74-ROS1* and non-*CD74 ROS1*) (*n* = 159), patients with non-reciprocal/reciprocal *ROS1* translocations (*n* = 9) had comparable ORR (77.8% vs 86.2%; *p* = 0.951; Table S3), DCR (100.0% vs 96.9%; *p* = 1.0; Table S4), and PFS (not reached vs 18.0 months; *p* = 0.116; Figure S1B) with crizotinib. Baseline clinicopathologic characteristics were similar for patients with single *ROS1* fusion and non-reciprocal/reciprocal *ROS1* translocations (Table S5). Table S6 lists the treatment outcomes on first-line crizotinib therapy of the 159 patients with single *ROS1* fusion grouped according to *ROS1* fusion partners. Table S7 and S8 list the detailed clinicopathologic features and clinical outcomes of the 13 patients with uncommon non-*CD74 ROS1* fusions and the 15 patients with non-reciprocal/reciprocal *ROS1* translocations, respectively.

### Impact of various molecular factors on crizotinib efficacy in ROS1-rearranged NSCLCs

Numerous reports have implicated the presence of other concomitant mutations in various genes including *TP53* in the poor response of *ALK*-rearranged NSCLC to crizotinib therapy [17, 18]; however, very limited reports have explored the clinical impact of other concomitant mutations on crizotinib therapy of *ROS1*-rearranged NSCLC. We then analyzed the genomic profile of 94 patients with *ROS1* fusions whose samples were sequenced using a panel with at least 168 genes and evaluable for survival outcomes on first-line crizotinib to investigate the correlation between the presence of certain concomitant mutations at baseline and crizotinib efficacy. For this analysis, concurrent mutations in only the 168 genes common across the gene panels used for NGS were analyzed. Among the 54 evaluable patients with *CD74-ROS1* fusions, 21 patients (38.9%) were detected with other concomitant mutations, with *TP53* (13.1%, 7/54) as the most common co-occurring mutation. Other co-occurring mutations in classic NSCLC oncogenic drivers, including *EGFR L858R* (n=1), *MET* amplification (*n* = 1), and *KRAS* G12D (*n* = 4), were detected from tissue samples of 6 patients. Meanwhile, among the 32 patients with non-*CD74 ROS1* fusions, 14 patients (43.7%) had other concomitant mutations, with *TP53* (18.8%, 6/32) as the most common. Concurrent mutations in classic NSCLC oncogenic drivers, including *MET* amplification (*n* = 3), were detected from tissue samples of 3 patients with non-*CD74 ROS1* fusion. The distribution of concurrent mutations detected among patients with *CD74-ROS1* fusions (*n* = 54) was not statistically different from those with non-*CD74 ROS1* fusions (*n* = 32) (38.9% vs. 43.7%; *p* = 0.774; Table S9).

**Fig. 3:**
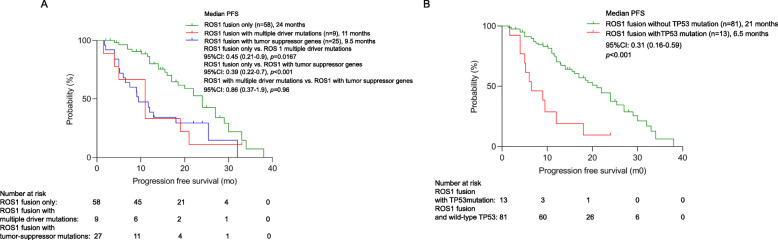
The presence of concomitant mutations is associated with poor prognosis. Kaplan-Meier survival curves illustrating the PFS for (**A**) patients with only *ROS1* fusions (green), for those with concurrent driver mutations (red), and with concomitant mutations in tumor suppressor genes (blue), and (**B**) patients with (red) and without (green) concomitant *TP53* mutations. The risk table below summarizes the number of patients included per time point

In general, as compared to patients with no co-occurring mutations (*n* = 58), PFS was significantly shorter for those who harbored concomitant driver mutations (*n* = 9) (11.0 months vs 24.0 months; *p* = 0.0167; Fig. 3A) or concomitant tumor suppressor genes (i.e., *TP53*, *RB1*, or *PTEN*) (*n* = 25) (9.5 months vs 24.0 months; *p* < 0.001; Fig. 3A). Patients with concomitant *TP53* mutations (*n* = 13) had significantly shorter PFS than those who had wild-type *TP53* (*n* = 81) (6.5 months vs. 21.0 months; *p* < 0.001; Fig. 3B). Multivariate analysis consistently demonstrate the impact of harboring concomitant *TP53* mutations (*p* = 0.028; hazard ratio: 0.41, 95% CI: 0.19–0.91) and harboring any concomitant mutations (*p* = 0.048; HR = 0.537, 95% CI: 0.290–0.995; Table S10) on PFS. These data suggest that the presence of concomitant mutations contributes to poor survival outcomes with first-line crizotinib therapy. Table S11 summarizes the clinical information of the 9 patients with concomitant driver mutations.

### Impact of baseline brain metastasis on crizotinib and chemotherapy efficacy

**Fig. 4:**
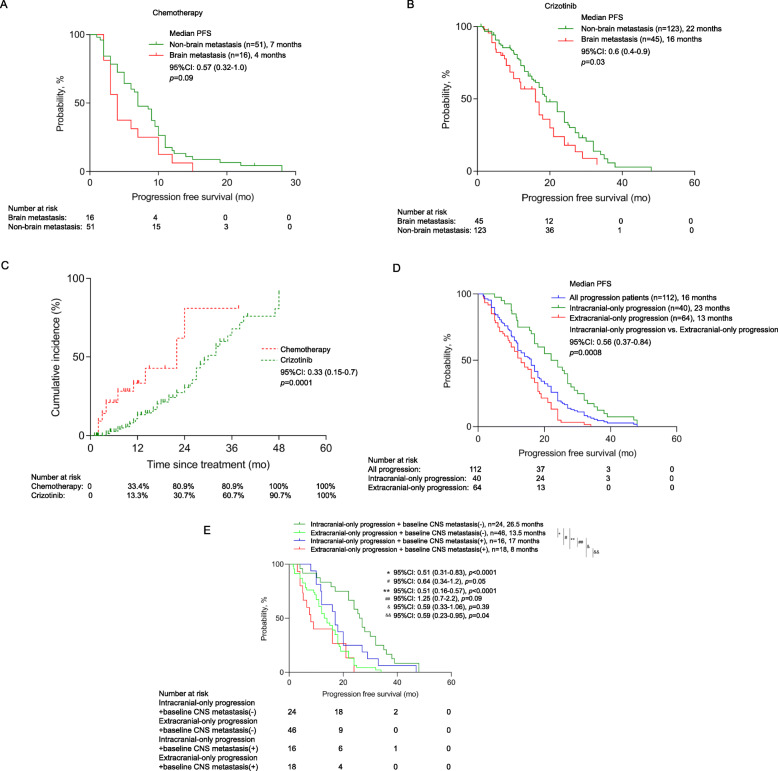
Patients with *ROS1*-rearranged NSCLC who had baseline brain metastasis still benefit from first-line crizotinib than chemotherapy. Kaplan-Meier survival curves illustrating the PFS for patients with *ROS1*-rearranged NSCLC who had non-brain metastasis (green) and brain metastasis (red) at baseline and received chemotherapy (**A**) and crizotinib (**B**) as first-line therapy. **C** Plot comparing the cumulative incidence of CNS progression in patients receiving crizotinib (green) and chemotherapy (red). **D**–**E** Crizotinib-treated patients with *ROS1*-rearranged NSCLC who experienced CNS only progression had a better prognosis than those who experienced non-CNS progression. D-E. Kaplan-Meier survival curves comparing the PFS of crizotinib-treated patients with *ROS1*-rearranged NSCLC who had non-CNS only progression (red), CNS only progression (green) and the whole cohort (blue) (**D**); **E** crizotinib-treated patients without baseline CNS metastasis who had CNS only progression (green) and non-CNS only progression (pink) and patients with baseline CNS metastasis who had CNS only progression (blue) and non-CNS only progression (red). The risk table below summarizes the number of patients included per time point

Since a substantial number of patients with advanced-stage NSCLC present with brain metastasis at initial presentation and that crizotinib is known to have limited penetrability to the blood-brain barrier, we assessed the clinical impact of baseline brain metastasis on crizotinib and chemotherapy efficacy. Among the 235 patients with advanced or locally advanced *ROS1*-rearranged NSCLCs, brain metastasis at presentation was detected from 58 patients (24.6%), which were detected using either MRI or CT scanning. Of them, 10 patients received whole-brain radiotherapy (WBRT) and 9 patients received stereotactic body radiotherapy (SBRT) for the management of their brain metastasis (Table 1). As compared to patients without brain metastasis at baseline, patients with baseline brain metastasis had a trend of shorter median PFS on first-line chemotherapy (4.0 months (*n* = 16) vs 7.0 months (*n* = 51); *p* = 0.09; Fig. 4A) and significantly shorter PFS on first-line crizotinib (16.0 months (*n* = 45) vs. 22.0 months (*n* = 123); *p* = 0.03; Fig. 4B). However, patients with brain metastasis at baseline had significantly longer median PFS with crizotinib therapy than with chemotherapy (16.0 months (*n* = 45) vs. 4.0 months (*n* = 16); *p* < 0.0001). The ORR (77.8% vs. 87.8%; *p* = 0.642) and DCR (95.6% vs. 97.5%; *p* = 0.933) with crizotinib therapy were comparable between patients with or without brain metastasis at baseline (Table S4). No difference in the presence of baseline brain metastasis was found among patients with *CD74-ROS1* and non-*CD74 ROS1* fusions (25.6% (23/90) vs. 27.5% (19/69); *p* = 0.830; Table 1). These findings indicate that despite the presence of brain metastasis at presentation, patients with *ROS1*-rearranged NSCLC still benefit more from crizotinib than chemotherapy.

### Central nervous system (CNS)-related progression is associated with better prognosis

We further investigated the pattern of disease progression and the prognostic role of CNS progression among the patients whose disease progressed from either chemotherapy or crizotinib therapy. Although the frequency of CNS progression was similar between the two treatment groups (crizotinib 28.6% (48/168) vs. chemotherapy 28.4% (19/67)), the median time of the appearance of CNS progression was significantly slower on crizotinib therapy than on chemotherapy (30.0 months vs 22.0 months; *p* < 0.001; Fig. 4C). Of the 168 patients who received crizotinib, disease progression was observed in 112 patients and the treatment of 56 patients was still ongoing as of the data cut-off. Of the relapsed patients, 35.7% (*n* = 40) patients had intracranial-only progression, 7.1% (*n* = 8) patients were simultaneously detected with intracranial and extracranial progression, while 57.1% (*n* = 64) patients had extracranial-only progression, including the enlargement of the primary lung lesion, and development of other extracranial metastatic lesions. Patients with both intracranial and extracranial progression (*n* = 8) were omitted from further analysis but their clinical details were summarized in Table S12. Patients who had intracranial-only progression (*n* = 40) achieved significantly longer PFS than those with extracranial-only progression (*n* = 64) (23.0 months vs. 13.0 months, *p* < 0.001; Fig. 4D). The baseline clinical characteristics were similar between the patients with intracranial only and extracranial only progression on crizotinib therapy (Table S13).

We further compared the survival outcomes of the patients based on the pattern of disease progression and the presence of baseline brain metastasis. Of the 40 crizotinib-treated patients who experienced intracranial-only progression, 16 patients had baseline CNS metastasis. Meanwhile, of the 64 crizotinib-treated patients with extracranial-only progression, 16 patients had baseline CNS metastasis (Table S13). The PFS were statistically different among the patients with or without baseline CNS metastasis who progressed with intracranial and extracranial metastasis (*p* = 0.007; Fig. 4E). Patients with baseline CNS metastasis who experienced intracranial-only progression with crizotinib therapy (*n* = 16) had significantly longer median PFS than those who experienced extracranial-only progression (*n* = 18) (17.0 months vs 8.0 months; *p* = 0.04; hazard ratio: 0.59, 95%CI: 0.23–0.95; Fig. 4E). Consistently, patients without baseline CNS metastasis who experienced intracranial-only progression with crizotinib therapy (*n* = 24) had longer median PFS than those who experienced extracranial-only progression (*n* = 46) (26.5 months vs 13.5 months; *p* < 0.001; hazard ratio: 0.51, 95%CI: 0.31–0.83; Fig. 4E). We found no difference in the pattern of progression with first-line crizotinib therapy among patients with *CD74-ROS1* and non-*CD74 ROS1* fusions (intracranial progression 41.0% vs. 46.8%; extracranial progression 59.0% vs. 53.2%; *p* = 0.679; Table 1). And there was also no difference for the local treatment (Table 1). Taken together, these data indicate that patients with CNS progression can still benefit from crizotinib therapy.

## Discussion

The current guidelines of the National Comprehensive Cancer Network recommend the use of ROS1 targeted inhibitors such as crizotinib and entrectinib as the preferred first-line treatment for *ROS1* rearrangement-positive NSCLC [19]. However, in real-world clinical practice, the use of targeted inhibitors can be restricted by the patient’s socio-economic status for molecular testing services and access to various treatment modalities. Hence, we aimed to investigate the efficacy of crizotinib and chemotherapy in patients with *ROS1*-rearranged NSCLC to gain a better understanding of the advantages of crizotinib therapy for this subset of patients in the real-world setting. In our retrospective study, we screened the molecular data of a large cohort of lung cancer patients from various hospitals in 4 provinces of China and revealed a prevalence of 2.1% for *ROS1* rearrangements among the Chinese patients with lung cancer. The prevalence we have derived from our cohort was consistent with the reported prevalence of 2.2% in another smaller cohort of Chinese patients with lung cancer [20, 21]. To the best of our knowledge, our study included the largest sample obtained from multiple centers, which reflects the actual prevalence of *ROS1* rearrangements in our population and could help identify effective therapeutic management of *ROS1*-rearranged NSCLCs.

Consistent with previous observations [5], chromosomal rearrangements involving *ROS1* in our cohort were mostly identified from younger patients with a median of 52 years old, females (63–66%), never smokers (75–76%), and with adenocarcinoma histology (97–98%). Our study has provided real-world clinical evidence on the efficacy of crizotinib in the first-line setting as reported in numerous clinical trials and other studies [8, 13, 14, 21–23]. The clinical outcomes we have observed from the 168 crizotinib-treated patients were consistent with another study that reported the better ORR, DCR, and PFS with first-line crizotinib as compared to chemotherapy for *ROS1*-rearranged NSCLC (ORR 80.0% vs 40.8%; DCR 90.0% vs. 71.4%; PFS 9.8 months vs 6.0 months) [21, 24]. Moreover, our results that demonstrated better survival outcomes with first-line crizotinib for patients with non-*CD74 ROS1* fusions than those who harbor *CD74-ROS1* fusions (21.0 months vs 17.0 months) were consistent with a prior report that demonstrated a PFS of 17.6 months and 12.6 months, respectively [12]. Meanwhile, another study reported an opposite trend of longer PFS with crizotinib for patients with *CD74-ROS1* than non-*CD74-ROS1* (20.1 months vs. 12.0 months) [24], while no difference in PFS with chemotherapy was observed between *CD74-ROS1* and non-*CD74-ROS1* (8.6 months vs. 4.3 months) [24]. Since some of the *ROS1* fusion partners are not as common as *CD74*, survival outcome data for patients with these fusion partners remains limited. Our results demonstrated a median PFS of 16.0 months for patients with *SDC4-ROS1*, 19.0 months each for patients with *EZR-ROS1* and *TPM3-ROS1*, and 22.0 months for patients with other uncommon non*-CD74 ROS1* fusion partners. Our study is the first to provide data on the treatment and survival outcomes on first-line crizotinib for patients harboring various novel/uncommon non-*CD74 ROS1* fusion partners as detailed in Table S7.

*TP53* is one of the most frequent concomitant mutations in NSCLC, and has been associated with poor prognosis of not only patients with *EGFR*-mutant NSCLC treated with EGFR-TKI but also patients with *ALK*-rearranged NSCLC treated with crizotinib or chemotherapy [17, 18, 25, 26]. *TP53* is a tumor suppressor gene and plays a critical role in regulating cell proliferation, and its loss-of-function promotes uncontrolled cell proliferation, tumor growth, and drug resistance [27–29]. Moreover, the detection of various concomitant mutations represents the molecular heterogeneity of the tumor, which could contribute to variable inhibitor response [30]. Our findings demonstrate poorer survival outcomes of patients with *ROS1*-rearranged NSCLC who harbored concomitant mutations in *TP53*, other tumor suppressor genes such as *PTEN* and *RB1*, and other oncogenic drivers such as *EGFR*, *MET* amplification, and *KRAS*. Our findings were consistent with the subgroup analysis of EUCROSS study on the shorter PFS of patients with *ROS1*-rearranged lung cancers with concomitant *TP53* mutations, treated with crizotinib than their wild-type counterpart [31].

Our study is limited by its retrospective nature; hence, some data are not available for analysis. Since the cohort with survival outcomes was based on the patients who submitted samples for NGS, inherent sampling bias might exist including the economic capability of the patients to submit samples for NGS and the decision on receiving chemotherapy or targeted therapy, which could limit our conclusion. Our study only analyzed the DNA-based NGS analysis of *ROS1* fusion variants and does not include in vitro functional assays or RNA-based analysis of patient samples to confirm the transcription of novel *ROS1* fusions or reciprocal/non-reciprocal *ROS1* translocations that we have identified from our cohort. Further in vitro experiments are necessary to provide functional evidence for these *ROS1* fusions.

## Conclusions

Our study provided real-world clinical evidence of the distinct efficacy of crizotinib among NSCLCs with various *ROS1* fusion partners including some novel non-*CD74 ROS1* fusions. Our findings demonstrate that as compared to chemotherapy, crizotinib is a better first-line therapy for patients with *ROS1*-rearranged NSCLC, with or without brain metastasis at presentation. Moreover, concurrent mutations may contribute to the poor clinical outcomes with crizotinib; hence, a better understanding of the patient’s mutation landscape is necessary for optimal treatment planning. Our study contributes to the understanding that baseline clinical and molecular factors could impact the survival outcomes of patients with locally advanced to advanced *ROS1*-rearranged NSCLC treated with first-line crizotinib.

## Supplementary Information

**Additional file 1:.** Figure S1. Kaplan Meier curves comparing the progression-free survival (expressed in months) of patients with (A) single CD74-ROS1 and various single non-CD74 ROS1 fusions; and (B) single ROS1 fusions and non-reciprocal/reciprocal ROS1 translocations. The risk table below summarizes the number of patients included per time point

**Additional file 2:.** Table S1. List of genes included in the 168-gene panel (Lung Plasma, Burning Rock Biotech). Table S2. Distribution of ROS1 fusions among the 220 patients with single ROS1 fusion and 15 patients with non-reciprocal/reciprocal ROS1 translocations. Table S3. Detailed breakpoint information for the non-reciprocal/reciprocal ROS1 translocations detected in the cohort. Table S4. Response to crizotinib of ROS1-rearranged lung cancers. Table S5. Clinicopathologic characteristics of the patients with single ROS1 fusions and non-reciprocal/reciprocal ROS1 translocations. Table S6. Treatment outcomes on first-line crizotinib therapy of the 159 patients with single ROS1 fusion grouped according to ROS1 fusion partners. Table S7. Detailed clinicopathologic characteristics and clinical outcomes of the 13 patients with uncommon non-CD74 ROS1 fusions. Table S8. Detailed clinicopathologic characteristics and clinical outcomes of the patients with non-reciprocal/reciprocal ROS1 translocations. Table S9. Comparison of concurrent mutation according to ROS1 mutation. Table S10. Cox regression analysis for progression-free survival (n=168). Table S11. Detailed clinicopathological characteristics and clinical outcomes of the 9 patients with concomitant driver mutations. Table S12. Detailed clinicopathological characteristics and clinical outcomes of the 8 patients with concomitant brain and non-brain progression on first-line crizotinib therapy. Table S13. Clinicopathological characteristics of the patients with brain progression and non-brain progression on first-line crizotinib therapy

## Data Availability

The datasets used and/or analyzed during the current study are available from the corresponding author upon reasonable request.

## Abbreviations

ARMS: Amplification refractory mutation system
CI: Confidence intervals
CNS: Central nervous system
CR: Complete response
CT: Computed tomography
DCR: Disease control rate
EGFR: Epidermal growth factor receptor
FISH: Fluorescence in situ hybridization
HR: Hazard ratio
MRI: Magnetic resonance imaging
NGS: Next-generation sequencing
NSCLC: Non-small-cell lung cancer
ORR: objective response rate
PFS: Progression-free survival
PD: Progressive disease
PR: Partial response
ROS1: ROS proto-oncogene-1
SD: Stable disease
TKI: Tyrosine kinase inhibitor
WBRT: Whole-brain radiotherapy
