# Supplementary material for: Clinical and molecular factors that impact the efficacy of first-line crizotinib in ROS1-rearranged non-small-cell lung cancer: a large multicenter retrospective study
Source: BMC Med. 2021 Sep 13;19:206. doi: 10.1186/s12916-021-02082-6 (PMC8436549; doi:10.1186/s12916-021-02082-6)
Supplement: Supplementary file 1 — Additional file 1:. Figure S1. Kaplan Meier curves comparing the progression-free survival (expressed in months) of patients with (A) single CD74-ROS1 and various single non-CD74 ROS1 fusions; and (B) single ROS1 fusions and non-reciprocal/reciprocal ROS1 translocations. The risk table below summarizes the number of patients included per time point [file 12916_2021_2082_MOESM1_ESM.pptx]

## Slide 1
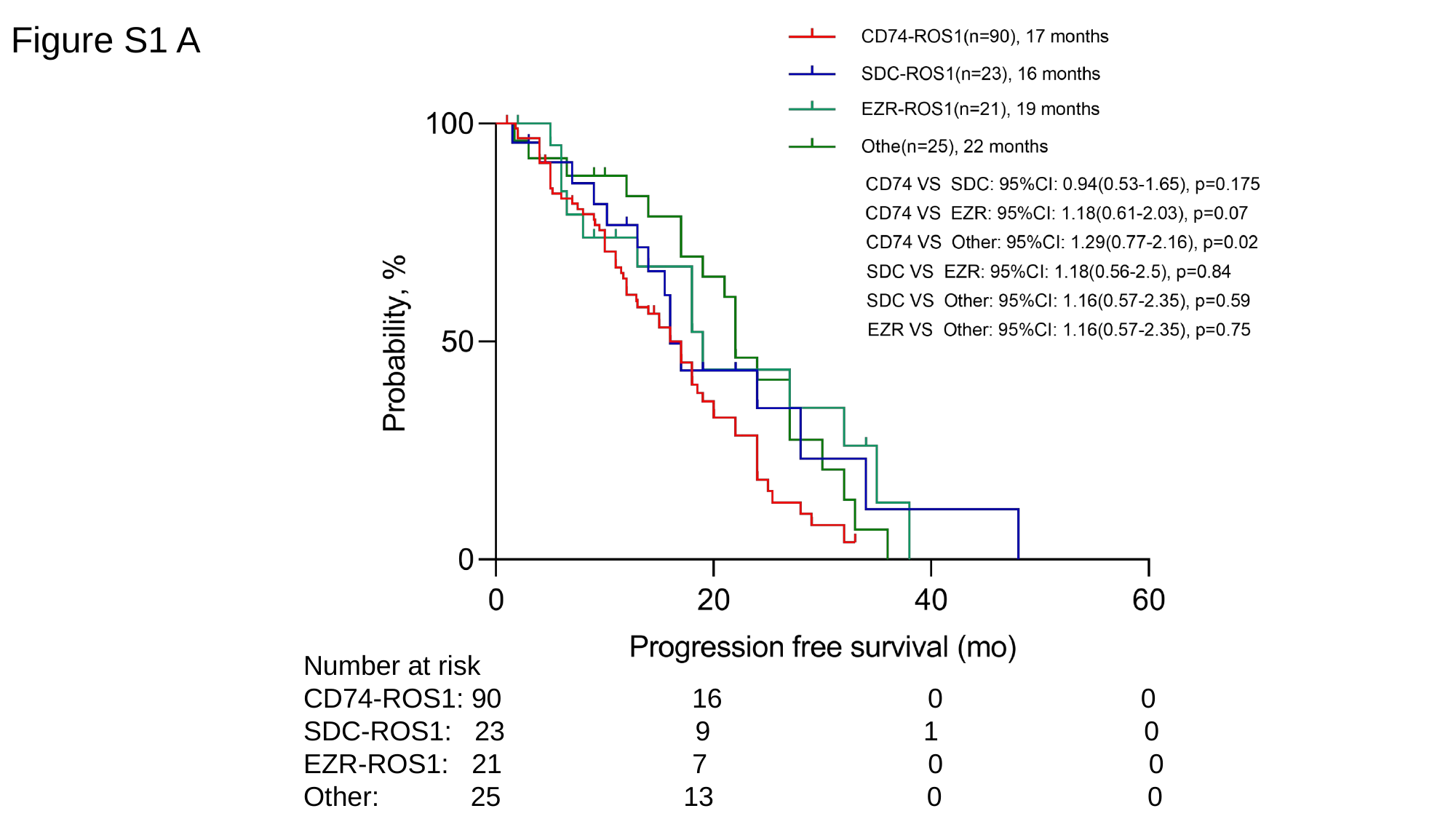

Figure S1 A
Number at risk
CD74-ROS1: 90 16 0 0
SDC-ROS1: 23 9 1 0
EZR-ROS1: 21 7 0 0
Other: 25 13 0 0

## Slide 2
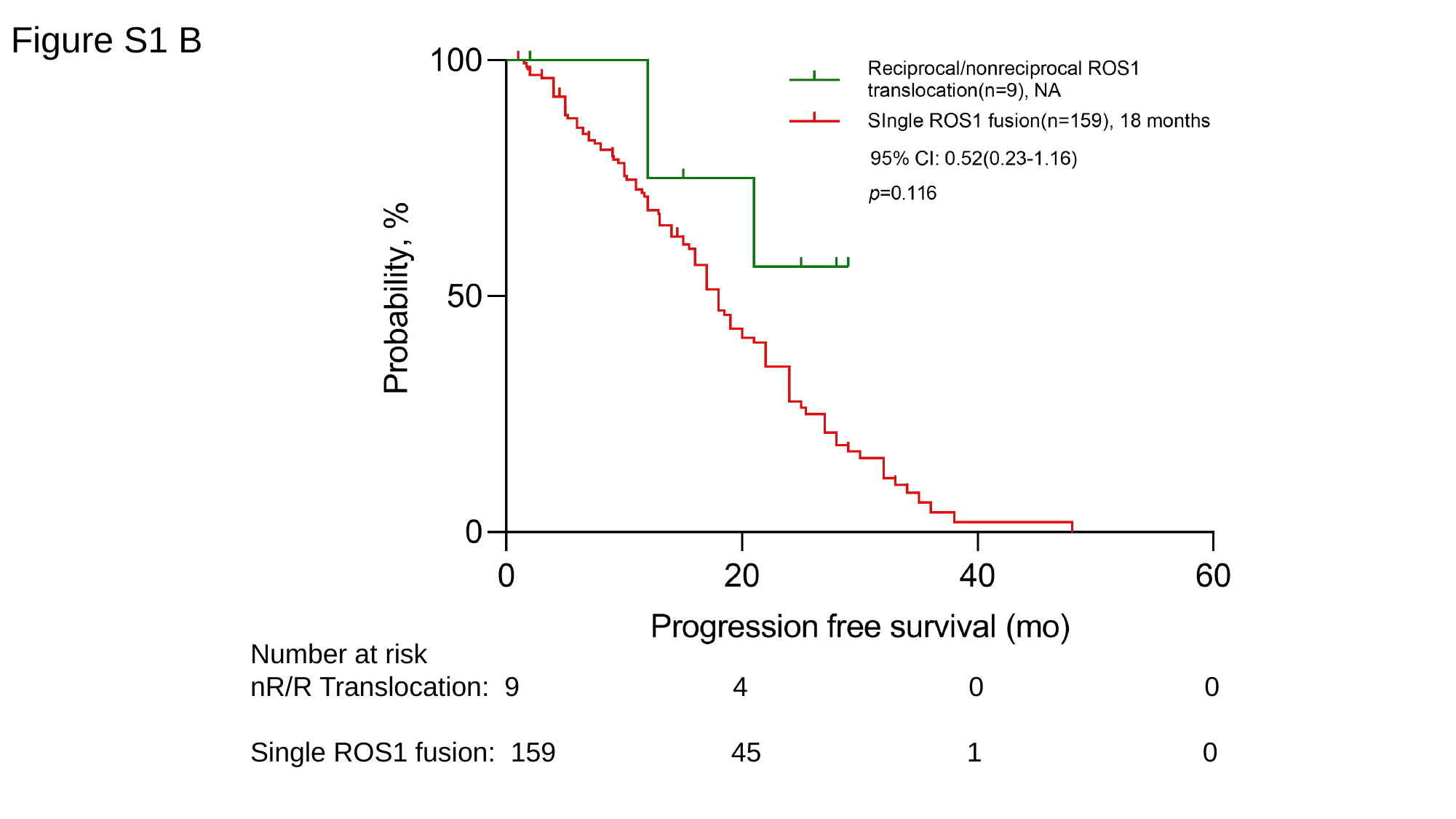

Figure S1 B
Number at risk
nR/R Translocation: 9 4 0 0
Single ROS1 fusion: 159 45 1 0
