# Supplementary material for: Clinical and molecular factors that impact the efficacy of first-line crizotinib in ROS1-rearranged non-small-cell lung cancer: a large multicenter retrospective study
Source: BMC Med. 2021 Sep 13;19:206. doi: 10.1186/s12916-021-02082-6 (PMC8436549; doi:10.1186/s12916-021-02082-6)
Supplement: Supplementary file 2 — Additional file 2:. Table S1. List of genes included in the 168-gene panel (Lung Plasma, Burning Rock Biotech). Table S2. Distribution of ROS1 fusions among the 220 patients with single ROS1 fusion and 15 patients with non-reciprocal/reciprocal ROS1 translocations. Table S3. Detailed breakpoint information for the non-reciprocal/reciprocal ROS1 translocations detected in the cohort. Table S4. Response to crizotinib of ROS1-rearranged lung cancers. Table S5. Clinicopathologic characteristics of the patients with single ROS1 fusions and non-reciprocal/reciprocal ROS1 translocations. Table S6. Treatment outcomes on first-line crizotinib therapy of the 159 patients with single ROS1 fusion grouped according to ROS1 fusion partners. Table S7. Detailed clinicopathologic characteristics and clinical outcomes of the 13 patients with uncommon non-CD74 ROS1 fusions. Table S8. Detailed clinicopathologic characteristics and clinical outcomes of the patients with non-reciprocal/reciprocal ROS1 translocations. Table S9. Comparison of concurrent mutation according to ROS1 mutation. Table S10. Cox regression analysis for progression-free survival (n=168). Table S11. Detailed clinicopathological characteristics and clinical outcomes of the 9 patients with concomitant driver mutations. Table S12. Detailed clinicopathological characteristics and clinical outcomes of the 8 patients with concomitant brain and non-brain progression on first-line crizotinib therapy. Table S13. Clinicopathological characteristics of the patients with brain progression and non-brain progression on first-line crizotinib therapy [file 12916_2021_2082_MOESM2_ESM.docx]

**Table S1.** The 168 panel genes list

| **gene** | **CNV** | **Fusion** | **SNV** | **gene** | **CNV** | **Fusion** | **SNV** | **gene** | **CNV** | **Fusion** | **SNV** | **gene** | **CNV** | **Fusion** | **SNV** | **gene** | **CNV** | **Fusion** | **SNV** |
| --- | --- | --- | --- | --- | --- | --- | --- | --- | --- | --- | --- | --- | --- | --- | --- | --- | --- | --- | --- |
| AKT1 | Y | Y | Y | EGFR | Y | Y | Y | JAK1 | Y | Y | Y | PIK3C3 | N | N | Y | TP53 | Y | N | Y |
| ALK | Y | Y | Y | EMSY | N | N | Y | JAK2 | Y | Y | Y | PIK3CA | Y | Y | Y | TP63 | N | N | Y |
| APC | Y | N | Y | EP300 | N | N | Y | KDM5A | N | N | Y | PIK3CG | Y | N | Y | TRIM58 | N | N | Y |
| AR | N | N | Y | EPHA3 | Y | N | Y | KDM6A | N | N | Y | PIK3R1 | Y | N | Y | TRPC5 | N | N | Y |
| ARID1A | Y | N | Y | EPHA5 | Y | N | Y | KDR | N | N | Y | PMS2 | Y | N | Y | U2AF1 | N | N | Y |
| ATM | N | N | Y | EPHA7 | N | N | Y | KEAP1 | Y | N | Y | POLD1 | N | N | Y | UGT1A1 | N | N | N |
| ATR | N | N | Y | EPHB1 | N | N | Y | KIT | Y | Y | Y | POLE | Y | N | Y | VEGFA | Y | N | Y |
| B2M | N | N | Y | ERBB2 | Y | Y | Y | KMT2D | N | N | Y | POM121L12 | N | N | Y | VEGFB | N | N | Y |
| BARD1 | N | N | Y | ERBB3 | N | Y | Y | KRAS | Y | N | Y | PPP2R1A | N | N | Y | VEGFC | N | N | Y |
| BCL2L11 | N | N | Y | ERBB4 | N | Y | Y | LRP1B | Y | N | Y | PRKDC | Y | N | Y | VHL | N | N | Y |
| BCOR | N | N | Y | ESR1 | N | Y | Y | MAP2K1 | N | N | Y | PTEN | Y | N | Y | YES1 | Y | N | Y |
| BLM | N | N | Y | FANCA | N | N | Y | MAP3K13 | N | N | Y | PTPRD | N | N | Y | NRG1 | N | Y | Y |
| BRAF | Y | Y | Y | FANCI | N | N | Y | MAX | N | N | Y | PTPRT | N | N | Y |  |  |  |  |
| BRCA1 | Y | N | Y | FAT3 | N | N | Y | MCL1 | N | N | Y | RAD50 | N | N | Y |  |  |  |  |
| BRCA2 | Y | N | Y | FBXW7 | Y | N | Y | MEN1 | N | N | Y | RAD51B | N | N | Y |  |  |  |  |
| BRINP3 | N | N | Y | FGF19 | Y | N | Y | MET | Y | Y | Y | RAD51C | N | N | Y |  |  |  |  |
| BRIP1 | N | N | Y | FGF3 | Y | N | Y | MLH1 | Y | N | Y | RAD51D | N | N | Y |  |  |  |  |
| CARD11 | N | N | Y | FGF4 | Y | N | Y | MRE11 | N | N | Y | RAD54L | N | N | Y |  |  |  |  |
| CASP8 | N | N | Y | FGFR1 | Y | Y | Y | MSH2 | Y | N | Y | RAF1 | N | Y | Y |  |  |  |  |
| CBL | N | N | Y | FGFR2 | Y | Y | Y | MSH6 | Y | N | Y | RARA | N | Y | Y |  |  |  |  |
| CCND1 | Y | N | Y | FGFR3 | Y | Y | Y | MTOR | Y | N | Y | RB1 | Y | N | Y |  |  |  |  |
| CCNE1 | Y | N | Y | FLT1 | N | N | Y | MUTYH | N | N | Y | RBM10 | N | N | Y |  |  |  |  |
| CD274 | Y | Y | Y | FLT3 | Y | N | Y | MYC | Y | Y | Y | RET | Y | Y | Y |  |  |  |  |
| CD74 | N | N | Y | FLT4 | N | N | Y | MYCN | Y | N | Y | RNF43 | Y | N | Y |  |  |  |  |
| CDH18 | N | N | Y | GATA2 | N | N | Y | NAV3 | N | N | Y | ROS1 | Y | Y | Y |  |  |  |  |
| CDK4 | Y | N | Y | GATA3 | Y | N | Y | NBN | N | N | Y | RUNX1 | N | N | Y |  |  |  |  |
| CDK6 | Y | N | Y | GRIN2A | N | N | Y | NF1 | Y | N | Y | SETD2 | N | N | Y |  |  |  |  |
| CDKN1A | N | N | Y | H3F3C | N | N | Y | NFE2L2 | Y | N | Y | SMAD4 | Y | N | Y |  |  |  |  |
| CDKN1B | N | N | Y | HGF | N | N | Y | NOTCH1 | N | Y | Y | SMARCA4 | Y | N | Y |  |  |  |  |
| CDKN2A | Y | N | Y | HIST1H1C | N | N | Y | NRAS | Y | N | Y | SOX2 | Y | N | Y |  |  |  |  |
| CHEK1 | N | N | Y | HIST1H3B | N | N | Y | NTRK1 | Y | Y | Y | SOX9 | N | N | Y |  |  |  |  |
| CHEK2 | N | N | Y | HIST1H3G | N | N | Y | NTRK2 | Y | Y | Y | SPOP | N | N | Y |  |  |  |  |
| CREBBP | N | N | Y | HRAS | N | N | Y | NTRK3 | Y | Y | Y | SPTA1 | N | N | Y |  |  |  |  |
| CSMD3 | Y | N | Y | IDH1 | N | N | Y | PAK5 | N | N | Y | SRC | N | N | Y |  |  |  |  |
| CTNNB1 | Y | N | Y | IDH2 | N | N | Y | PALB2 | N | N | Y | STAG2 | N | N | Y |  |  |  |  |
| CYP2D6 | N | N | N | IGF2 | N | N | Y | PARP1 | N | N | Y | STK11 | Y | N | Y |  |  |  |  |
| DIS3 | N | N | Y | IKZF1 | N | N | Y | PDGFRA | Y | Y | Y | TBX3 | N | N | Y |  |  |  |  |
| DNMT3A | N | N | Y | IL7R | N | N | Y | PDGFRB | N | Y | Y | TERT | N | Y | Y |  |  |  |  |
| DPYD | N | N | N | INHBA | N | N | Y | PIK3C2G | N | N | Y | TGFBR2 | N | N | Y |  |  |  |  |

**Table S2.** Distribution of *ROS1* fusions among the 220 patients with single *ROS1* fusion and 15 patients with non-reciprocal/reciprocal *ROS1* translocations

| Single *ROS1* fusion | n=220(93.6%) | % | Non-reciprocal/Reciprocal *ROS1* Translocation | n=15(6.4%) | % |
| --- | --- | --- | --- | --- | --- |
| *CD74-ROS1* | 130 | 59.1% | *CD74-ROS1+ROS1-PUM1** | 1 | 6.7% |
| *SDC4-ROS1* | 29 | 13.2% | *CD74-ROS1+ROS1-HMGXB3** | 2 | 13.3% |
| *EZR-ROS1* | 25 | 11.4% | *CD74-ROS1+ROS1-MRAS** | 1 | 6.7% |
| *TPM3-ROS1* | 16 | 7.3% | *CD74-ROS1+ROS1-ADGRV1** | 1 | 6.7% |
| *SLC34A2-ROS1* | 6 | 2.8% | *CD74-ROS1+ROS1-SPEF2** | 1 | 6.7% |
| *LRIG3-ROS1* | 3 | 1.4% | *CD74-ROS1+SPOCK1-ROS1** | 1 | 6.7% |
| *CCDC6-ROS1* | 2 | 0.9% | *EZR-ROS1+CD44-ROS1** | 1 | 6.7% |
| *MYH9-ROS1** | 2 | 0.9% | *CD74-ROS1+SDC4-ROS1+SLC34A2-ROS1** | 1 | 6.7% |
| *ZCCHC8-ROS1* | 2 | 0.9% | *EZR-ROS1+ROS1-XPNPEP1** | 1 | 6.7% |
| *AQP4-ROS1** | 1 | 0.45% | *EZR-ROS1+ROS1-BTBD9** | 1 | 6.7% |
| *CTNND2-ROS1** | 1 | 0.45% | *SDC4-ROS1+ROS1-LRRK2** | 1 | 6.7% |
| *GOPC-ROS1* | 1 | 0.45% | *SDC4-ROS1+ROS1-C19orf54** | 1 | 6.7% |
| *PHACTR3-ROS1** | 1 | 0.45% | *ZNF397-ROS1+ROS1-QKI** | 1 | 6.7% |
| *PTM-ROS1** | 1 | 0.45% | *RBPMS-ROS1+ROS1-GTF2E2** | 1 | 6.7% |
|  |  |  |  |  |  |

*Novel finding

**Table S3.** Detailed breakpoint information for the non-reciprocal/reciprocal *ROS1* translocations detected in the cohort

| Patient number | *ROS1* fusion | Chromosome | Variation type | Breakpoint | HGVS | Gene_L | Gene_R | Retention of kinase domain |
| --- | --- | --- | --- | --- | --- | --- | --- | --- |
| NO1 | *CD74-ROS1* | 6 | Chromosomal translocation | Intron6_Intron33 | CD74-ROS1(C6:R34) | ROS1 | CD74 | Retained |
| NO1 | *MRAS-ROS1* | 6 | Chromosomal translocation | Intron1_Intron33 | MRAS-ROS1(M5'UTR:R33) | MRAS | ROS1 | Absent |
| NO2 | *CD74-ROS1* | 6 | Chromosomal translocation | Intron6_Intron33 | CD74-ROS1(C6:R34) | ROS1 | CD74 | Retained |
| NO2 | *PUM1-ROS1* | 6 | Chromosomal translocation | Intron13_Intron33 | PUM1-ROS1(P13:R33) | PUM1 | ROS1 | Absent |
| NO3 | *ROS1-ADGRV1* | 6 | Chromosomal translocation | Intron33_Intron83 | ROS1-ADGRV1(R33:A84) | ADGRV1 | ROS1 | Absent |
| NO3 | *CD74-ROS1* | 6 | Chromosomal translocation | Intron6_Intron33 | CD74-ROS1(C6:R34) | ROS1 | CD74 | Retained |
| NO4&11 | *EZR-ROS1* | 6 | Deletion | Intron10_Intron33 | EZR-ROS1(E10:R34) | ROS1 | EZR | Retained |
| NO4&11 | *XPNPEP1-ROS1* | 6 | Chromosomal translocation | Intron5_Intron33 | XPNPEP1-ROS1(X5:R33) | ROS1 | XPNPEP1 | Absent |
| NO5&16 | *EZR-ROS1* | 6 | Deletion | Intron10_Intron33 | EZR-ROS1(E10:R34) | ROS1 | EZR | Retained |
| NO5&16 | *BTBD9-ROS1* | 6 | Inversion | Intron1_Intron33 | BTBD9-ROS1(B5'UTR: R33) | BTBD9 | ROS1 | Absent |
| NO6 | *SDC4-ROS1* | 6 | Chromosomal translocation | Intron2_Intron31 | SDC4-ROS1(S2:R32) | ROS1 | SDC4 | Retained |
| NO6 | *C19orf54-ROS1* | 6 | Chromosomal translocation | Intron1_Intron31 | C19orf54-ROS1(C2:R32) | ROS1 | C19orf54 | Retained |
| NO7 | *intergenic (MAPRE2, ZNF397)-ROS1* | 6 | Chromosomal translocation | intergenic_Intron32 | intergenic(MAPRE2,ZNF397)-ROS1(R33) | ROS1 | intergenic(MAPRE2,ZNF397) | Retained |
| NO7 | *QKI-ROS1* | 6 | Tandem duplication | Intron4_Intron32 | QKI-ROS1(Q4:R32) | ROS1 | QKI | Absent |
| NO8 | *GTF2E2-ROS1* | 6 | Chromosomal translocation | Intron6_Intron34 | GTF2E2-ROS1(G6:R34) | ROS1 | GTF2E2 | Absent |
| NO8 | *RBPMS-ROS1* | 6 | Chromosomal translocation | Exon7_Intron34 | RBPMS-ROS1(R6:R35) | ROS1 | RBPMS | Retained |
| NO9 | *EZR-ROS1* | 6 | Deletion | Intron10_Intron32 | EZR-ROS1(E10:R33) | ROS1 | EZR | Retained |
| NO9 | *CD44-ROS1* | 6 | Chromosomal translocation | Intron2_Intron32 | CD44-ROS1(C2:R32) | CD44 | ROS1 | Absent |
| NO10 | *SLC34A2-ROS1* |  | Chromosomal translocation | Exon7_Intron34 | SLC34A2-ROS1(S6:R33) | SLC34A2 | ROS1 | Retained |
| NO10 | *CD74-ROS1* | 6 | Chromosomal translocation | Intron6_Intron33 | CD74-ROS1(C6:R34) | ROS1 | CD74 | Retained |
| NO10 | *SDC4-ROS1* | 6 | Chromosomal translocation | Intron2_Intron31 | SDC4-ROS1(S2:R32) | ROS1 | SDC4 | Retained |
| NO12&17 | *CD74-ROS1* | 6 | Chromosomal translocation | Intron6_Intron33 | CD74-ROS1(C6:R34) | ROS1 | CD74 | Retained |
| NO12&17 | *HMGXB3-ROS1* |  | Chromosomal translocation | Intron2_Intron32 | HMGXB3-ROS1(H2:R32) | HMGXB3 | ROS1 | Absent |
| NO13 | *SDC4-ROS1* | 6 | Chromosomal translocation | Intron2_Intron31 | SDC4-ROS1(S2:R32) | ROS1 | SDC4 | Retained |
| NO13 | *LRRK2-ROS1* |  | Chromosomal translocation | Intron10_Intron33 | LRRK2-ROS1(L9:R32) | LRRK2 | ROS1 | Absent |
| NO14 | *CD74-ROS1* | 6 | Chromosomal translocation | Intron6_Intron33 | CD74-ROS1(C6:R34) | ROS1 | CD74 | Retained |
| NO14 | *SPEF2-ROS1* |  | Chromosomal translocation | Intron6_Intron33 | SPEF2-ROS1(S5:R34) | SPEF2 | ROS1 | Absent |
| NO15 | *CD74-ROS1* | 6 | Chromosomal translocation | Intron6_Intron33 | CD74-ROS1(C6:R34) | ROS1 | CD74 | Retained |
| NO15 | *ROS1-SPOCK1* |  | Chromosomal translocation | Intron2_Intron3 | SPOCK1-ROS1(S2:R34) | SPOCK1 | ROS1 | Absent |

**Table S4.** Response to crizotinib of *ROS1*-rearranged lung cancers

| Response |  | *ROS1* fusion partner | | | | Baseline brain metastasis | | |
| --- | --- | --- | --- | --- | --- | --- | --- | --- |
|  | All (n=168, n, %) | *CD74-ROS1* (n=90, n, %) | non-*CD74-ROS1* (n=69, n, %) | Non-reciprocal/reciprocal *ROS1* translocation (n=9, n, %) | *p* | Brain metastasis (n=45, n, %) | Non-brain metastasis (n=123, n, %) | *p* |
| Complete response | 1 (0.6) | 1 (1) | 0 (0) | 0 (0) |  | 0 (0) | 1 (0.8) |  |
| Partial response | 143 (85.1) | 79 (87.8) | 57(82.6) | 7(77.8) |  | 35 (77.8) | 108 (87.8) |  |
| Stable disease | 19 (11.3) | 7 (7.8) | 10 (14.5) | 2(22.3) |  | 8 (15.5) | 11 (8.9) |  |
| Progressive disease | 5 (2.9) | 3 (3.4) | 2 (2.9) | 0 (0) |  | 2 (6.7) | 3 (2.5) |  |
| Overall response rate | 85.7% | 88.9% | 82.6% | 77.8% | 0.951 | 77.8% | 87.8% | 0.642 |
| Disease control rate | 97.1% | 96.7% | 97.1% | 100.0% | 0.997 | 95.6% | 97.5% | 0.933 |

**Table S5.** Clinicopathologic characteristics of the patients with single *ROS1* fusions and non-reciprocal/reciprocal *ROS1* translocations

| Clinicopathological characteristics | Single *ROS1* fusion (n=159) | Non-reciprocal/reciprocal *ROS1* translocation (n=9) | *p*-value |  |
| --- | --- | --- | --- | --- |
|  |  |  |  |  |
| Age-yr (median, range) | 51(27-79) | 54(37-74) | 0.713 |  |
| Sex |  |  | 0.330 |  |
| Male | 61(38.4) | 2(22.2) |  |  |
| Female | 98(61.6) | 7(77.8) |  |  |
| Clinical stage |  |  | 0.501 |  |
| III | 9(5.7) | 1(11.1) |  |  |
| IV | 150(94.3) | 8(88.9) |  |  |
| ECOG PS score |  |  | 0.467 |  |
| 0-1 | 153(96.2) | 9(100) |  |  |
| ≥2 | 6(3.8) | 0(0) |  |  |
| Tumor histology |  |  | 0.735 |  |
| Adenocarcinoma | 158(99.4) | 9(100) |  |  |
| Squamous cell carcinoma | 1(0.6) | 0(0) |  |  |
| Presence of brain metastasis at baseline |  |  | 0.777 |  |
| Present | 46(28.9) | 3（33.3) |  |  |
| Absent | 113(71.10 | 6(66.7) |  |  |
| Methods used for evaluating brain metastasis |  |  | 0.313 |  |
| CT | 62(39) | 2(22.2) |  |  |
| MRI | 97(61) | 7(77.8) |  |  |
| Local therapy received for management of brain metastasis |  |  | 0.562 |  |
| None | 33(71.8) | 2(66.7) |  |  |
| WBRT | 7(15.2) | 1(33.3) |  |  |
| SBRT | 6(13) | 0(0) |  |  |
| Site of progression |  |  | 0.233 |  |
| Brain | 47(29.6) | 1(11.1） |  |  |
| Non-brain | 112(70.4) | 8(88.9) |  |  |

**Table S6**. Treatment outcomes on first-line crizotinib therapy of the 159 patients with single *ROS1* fusion grouped according to *ROS1* fusion partners

| Various *ROS1* fusions | Complete response  n(%) | Partial response n(%) | Stable disease n(%) | Progressive disease n(%) | Objective response rate | Disease control rate |
| --- | --- | --- | --- | --- | --- | --- |
| *CD74-ROS1* (n=90) | 1(1.11%) | 83(92.2%) | 5(55.6%) | 1(1.11%) | 93.3% | 98.9% |
| *SDC4-ROS1* (n=23) | 0(0.0%) | 21(91.3%) | 1(4.3%) | 1(4.3%) | 91.3% | 95.7% |
| *EZR-ROS1* (n=21) | 0(0.0%) | 18(85.7%) | 3(14.3%) | 0(0.0%) | 85.7% | 14.3% |
| *TPM3-ROS1* (n=12) | 0(0.0%) | 11(91.7%) | 1(8.3%) | 0(0.0%) | 91.7% | 100.0% |
| *SLC34A2-ROS1* (n=3) | 0(0.0%) | 3(100.0%) | 0(0.0%) | 0(0.0%) | 100.0% | 100.0% |
| *LRIG3-ROS1* (n=3) | 0(0.0%) | 2(66.7%) | 0(0.0%) | 1(33.3%) | 66.7% | 66.7% |
| *MYH9-ROS1* (n=1) | 0(0.0%) | 1(100.0%) | 0(0.0%) | 0(0.0%) | 100.0% | 100.0% |
| *CCDC6-ROS1* (n=1) | 0(0.0%) | 1(100.0%) | 0(0.0%) | 0(0.0%) | 100.0% | 100.0% |
| *AQP4-ROS1* (n=1) | 0(0.0%) | 1(100.0%) | 0(0.0%) | 0(0.0%) | 100.0% | 100.0% |
| *CTNND2-ROS1* (n=1) | 0(0.0%) | 1(100.0%) | 0(0.0%) | 0(0.0%) | 100.0% | 100.0% |
| *PHACTR3-ROS1* (n=1) | 0(0.0%) | 1(100.0%) | 0(0.0%) | 0(0.0%) | 100.00% | 100.00% |
| *GOPC-ROS1* (n=1) | 0(0.0%) | 0(0.0%) | 0(0.0%) | 1(100.0%) | 0.00% | 0.00% |
| *PTM-ROS1* (n=1) | 0(0.0%) | 1(100.0%) | 0(0.0%) | 0(0.0%) | 100.00% | 100.00% |

**Table S7.** Detailed clinicopathologic characteristics and clinical outcomes of the 13 patients with uncommon non-*CD74 ROS1* fusions

| Patient number | *ROS1* fusion | Age | Sex | Tumor histology | Clinical stage | Baseline metastasis (brain or non-brain) | Local therapy for brain metastasis | Treatment regimen received | Best response | PFS (months) | Site of progression |
| --- | --- | --- | --- | --- | --- | --- | --- | --- | --- | --- | --- |
| 1 | *CTNND2-ROS1* | 51 | Male | Adenocarcinoma | IV | Brain | WBRT | Crizotinib | PR | 27 | brain |
| 2 | *LRIG3-ROS1* | 33 | Male | Adenocarcinoma | IV | Brain | SBRT | Crizotinib | PR | 33 | brain |
| 3 | *PHACTR3-ROS1* | 61 | Male | Adenocarcinoma | III | Non-brain | no | Crizotinib | PR | 22 | Ongoing |
| 4 | *MYH9-ROS1* | 47 | Female | Adenocarcinoma | IV | Non-brain | no | Crizotinib | PR | 24 | Ongoing |
| 5 | *LRIG3-ROS1* | 47 | Male | Adenocarcinoma | IV | Non-brain | no | Crizotinib | PD | 1.7 | non-brain |
| 6 | *SLC34A2-ROS1* | 44 | Female | Adenocarcinoma | IV | Non-brain | no | Crizotinib | PR | 9 | Ongoing |
| 7 | *LRIG3-ROS1* | 55 | Female | Adenocarcinoma | IV | Brain | no | Crizotinib | PR | 24 | Ongoing |
| 8 | *PTM-ROS1* | 43 | Female | Adenocarcinoma | IV | Non-brain | no | Crizotinib | PR | 17 | non-brain |
| 9 | *AQP4-ROS1* | 61 | Male | Adenocarcinoma | IV | Brain | no | Crizotinib | PR | 21 | non-brain |
| 10 | *SLC34A2-ROS1* | 52 | Female | Adenocarcinoma | IV | Non-brain | no | Crizotinib | PR | 22 | non-brain |
| 11 | *CCDC6-ROS1* | 33 | Female | Adenocarcinoma | IV | Non-brain | no | Crizotinib | PR | 22 | brain |
| 12 | *MYH9-ROS1* | 55 | Female | Adenocarcinoma | IV | Non-brain | no | Crizotinib | PR | 9 | Ongoing |
| 13 | *SLC34A2-ROS1* | 55 | Female | Adenocarcinoma | IV | Non-brain | no | Crizotinib | PR | 32 | brain |

**Table S8.** Detailed clinicopathologic characteristics and clinical outcomes of the patients with non-reciprocal/reciprocal *ROS1* translocations

| Patient number | Age (years) | Sex | Smoking history | ECOG PS | Clinical stage | Brain metastasis | Histology | Driver oncogene mutation | First-line treatment |
| --- | --- | --- | --- | --- | --- | --- | --- | --- | --- |
| NO.1 | 58 | Male | Former | 0 | IV | 1 | Adenocarcinoma | *CD74-ROS1+ROS1-MRAS* | Pemetrexed plus Carboplatin |
| NO.2 | 52 | Female | Never | 1 | IV | 0 | Adenocarcinoma | *CD74-ROS1+ROS1-PUM1* | Pemetrexed plus Carboplatin |
| NO.3 | 47 | Female | Never | 1 | III | 0 | Adenocarcinoma | *CD74-ROS1+ROS1-ADGRV1* | Crizotinib |
| NO.4 | 54 | Female | Never | 1 | III | 0 | Adenocarcinoma | *EZR-ROS1+ROS1-XPNPEP1* | Pemetrexed plus Carboplatin |
| NO.5 | 54 | Male | Former | 1 | IV | 1 | Adenocarcinoma | *EZR-ROS1+ROS1-BTBD9* | Crizotinib |
| NO.6 | 66 | Female | Never | 0 | IV | 0 | Adenocarcinoma | *SDC4-ROS1-ROS1-C19orf54* | Crizotinib |
| NO.8 | 38 | Female | Never | 1 | III | 0 | Adenocarcinoma | *RBPMS-ROS1+ROS1-GTF2E2* | Crizotinib |
| NO.9 | 38 | Female | Never | 1 | IV | 0 | Adenocarcinoma | *EZR-ROS1+ROS1-CD44* | Crizotinib |
| NO.10 | 37 | Female | Never | 1 | IV | 1 | Adenocarcinoma | *SLC34A2-ROS1+CD74-ROS1+SDC4-ROS1* | Crizotinib |
| NO.11 | 74 | Female | Never | 1 | IV | 1 | Adenocarcinoma | *EZR-ROS1+XPNPEP1-ROS1* | Crizotinib |
| NO.12 | 62 | Male | Former | 1 | IV | 1 | Adenocarcinoma | *CD74-ROS1+ROS1-HMGXB3* | Crizotinib |
| NO.13 | 59 | Male | Former | 1 | IV | 1 | Adenocarcinoma | *SDC4-ROS1+ROS1-LRRK2* | Pemetrexed plus Carboplatin |
| NO.14 | 42 | Male | Former | 0 | IV | 0 | Adenocarcinoma | *CD74-ROS1+ROS1-SPEF2* | Pemetrexed plus Carboplatin |
| NO.15 | 71 | Female | Former | 1 | IV | 0 | Adenocarcinoma | *CD74-ROS1+ROS1-SPOCK1* | Crizotinib |
| NO.17 | 56 | Female | Former | 0 | IV | 0 | Adenocarcinoma | *CD74-ROS1+ROS1-HMGXB3* | Pemetrexed plus Carboplatin |

**Table S8.** Detailed clinicopathologic characteristics and clinical outcomes of patients with non-reciprocal/reciprocal *ROS1* translocations(continue1)

| % maximum tumor reduction | PFS1 | Adverse event1 | Progression model1 | Resistance mechanism1 | Second line treatment | % maximum tumor reduction2 | PFS2 | Adverse event2 | Progression model2 | Resistance mechanism2 |
| --- | --- | --- | --- | --- | --- | --- | --- | --- | --- | --- |
| -35% | 15 | / | non-brain | / | Crizotinib | -85% | 19 | / | non-brain | / |
| 10% | 4 | / | non-brain | / | Crizotinib | -65% | 7.5 | / | non-brain | / |
| -70% | 29+ | / |  |  |  |  |  |  |  |  |
| -45% | 14 | / | brain | / | Crizotinib | 15% | 3 | / | / | / |
| -85% | 28+ | / |  |  |  |  |  |  |  |  |
| -37% | 12 | / | brain | / | Loratinib+R | -95% | 11+ | / |  |  |
| / | / | / |  |  |  |  |  |  |  |  |
| -49% | 15+ | / |  |  |  |  |  |  |  |  |
| -39% | 2+ | / |  |  |  |  |  |  |  |  |
| -55% | 15+ | / |  |  |  |  |  |  |  |  |
| -67% | 25+ | / |  |  |  |  |  |  |  |  |
| -90% | 21 | / | non-brain | / | Crizotinib | 70% | 2 | / | brain | PIK3CA;PIK3R2 |
| -50% | 12 | / | non-brain | / | Crizotinib | -10% | 3.5 | / | brain | / |
| 80% | 2 | / | brain | / | Crizotinib | -50% | 8 | / |  |  |
| -70% | 12 | / | non-brain | / | Crizotinib | -90% | 12+ | / |  |  |
| / | / | / |  |  |  |  |  |  |  |  |
| -60% | 8.5 | / | non-brain | / | Crizotinib | 100% | 1.4 | / | non-brain | / |

**Table S8.** Detailed clinicopathologic characteristics and clinical outcomes of patients with non-reciprocal/reciprocal *ROS1* translocations(continue2)

| Third line treatment | % maximum tumor reduction3 | PFS3 | Adverse event3 | Progression model3 | Resistance mechanism3 | Fouth line treatment | % maximum tumor reduction4 | PFS4 | Adverse event4 | Progression model4 | Resistance mechanism4 | ∑ PFS (months) | OS (months) |
| --- | --- | --- | --- | --- | --- | --- | --- | --- | --- | --- | --- | --- | --- |
| Crizotinib+SBRT | -10% | 16 | / | lung progression | / | Lorlatinib | -10% | 5 | / | lung progression | / |  | 65 |
|  |  |  |  |  |  |  |  |  |  |  |  |  | 14 |
|  |  |  |  |  |  |  |  |  |  |  |  |  | 29+ |
| Crizotinib+SBRT | -20% | 4+ | / |  |  |  |  |  |  |  |  |  | 25 |
|  |  |  |  |  |  |  |  |  |  |  |  |  | 28+ |
|  |  |  |  |  |  |  |  |  |  |  |  |  | 23+ |
|  |  |  |  |  |  |  |  |  |  |  |  |  |  |
|  |  |  |  |  |  |  |  |  |  |  |  |  | 15+ |
|  |  |  |  |  |  |  |  |  |  |  |  |  | 2+ |
|  |  |  |  |  |  |  |  |  |  |  |  |  | 15+ |
|  |  |  |  |  |  |  |  |  |  |  |  |  | 25+ |
| Lorlatinib | -50% | 3+ | / |  |  |  |  |  |  |  |  |  | 25+ |
| Lorlatinib+SBRT | -40% | 4 | / |  |  |  |  |  |  |  |  |  | 24 |
|  |  |  |  |  |  |  |  |  |  |  |  |  | 10 |
|  |  |  |  |  |  |  |  |  |  |  |  |  | 24 |
|  |  |  |  |  |  |  |  |  |  |  |  |  |  |
| Paclitaxel plus Carboplatin | 10% | 4.8 | / | / | / | Crizotinib | -15% | 4.8 | / | / | / |  | 36 |

**Table S9**. Comparison of concurrent mutation according to *ROS1* mutation

| Concomitant mutated genes | All (n=94) | *ROS1* fusion partner | | | *p*-value | Baseline brain metastasis | | *p*-value |
| --- | --- | --- | --- | --- | --- | --- | --- | --- |
|  |  | Single *CD74-ROS1* (n=54,57.4% ) | Single non-*CD74 ROS1* (n=32, 34.0%) | Non-reciprocal/reciprocal *ROS1* translocation (n=8, 8.6%) |  | Brain metastasis (n=26, 27.6%) | Non-brain metastasis (n=68, 72.4%) |  |
| *ROS1* fusion + *TP53* mutation | 13(13.8%) | 7(13.1%) | 6(18.8%) | 0(0) | 0.536 | 4(15.4%) | 9(13.2%) | 0.814 |
| *ROS1* fusion + multiple driver genes | 11(11.7%) | 6(11%) | 5(15.6%) | 0(0) | 0.448 | 4(15.4%) | 10(14.7%) | 0.943 |
| *ROS1* fusion + tumor suppressor genes | 25(26.6%) | 14(25.9%) | 10(21.2%) | 1(12.5%) | 0.691 | 6(23%) | 16((23.5%) | 0.974 |
| With concomitant mutations | 36(38.3) | 21(38.9%) | 14(43.7%) | 1(12.5%) | 0.774 | 10(38.4%) | 26 (40.6%) | 0.981 |
| Without concomitant mutation | 58(61.7%) | 33(61.1%) | 18(56.3%) | 7(87.5%) | 0.821 | 16(61.6%) | 42(59.4%) | / |

**Table S10.** Cox regression analysis for progression-free survival (n=168)

| **Variable** | **Univariate Analysis** | **Multivariate Analysis** | |
| --- | --- | --- | --- |
|  | ***P* value** | **Hazard Ratio (95% CI)** | ***P* value** |
| **Sex** (male vs. female) | 0.497 |  |  |
| **Age** (≥57 vs. <57 years) | 0.296 |  |  |
| **Smoking status** (smoker/former smoker vs. non-smoker) | 0.424 |  |  |
| **Brain metastasis** (present/with vs. absent/without) | 0.030 | 0.797(0.454-1.399) | 0.428 |
| **TNM stage (IV vs. IIIB-C)** | 0.254 |  |  |
| **Pathology (Squamous carcinoma vs. adenocarcinoma)**  ***ROS1* fusion type (CD74 vs. non-CD74)**  **Concomitant *TP53* mutation (mutant vs. wild type)**  **Concomitant mutations (yes vs.no)** | 0.874  0.008  ＜0.001  ＜0.001 | 1.390(0.800-2.417)  0.413(0.188-0.908)  0.537(0.290-0.995) | 0.243  0.028  0.048 |

**Table S11**. Detailed clinicopathological characteristics and clinical outcomes of the 9 patients with concomitant driver mutations.

| Patient number | Age | Sex | Clinical stage | Histology | ROS1 fusion | Concomitant mutation | Abundance (%) | Best response to crizotinib | PFS (months) |
| --- | --- | --- | --- | --- | --- | --- | --- | --- | --- |
| NO1 | 52 | Female | IV | Adenocarcinoma | SDC4-ROS1 | MET amplification | 5.68 | PD | 1.5 |
| NO2 | 49 | Male | IV | Adenocarcinoma | CD74-ROS1 | MET amplification | 7.89 | PR | 15.8 |
| NO3 | 48 | Female | IV | Adenocarcinoma | CD74-ROS1 | EGFR L858R | 23.91 | PR | 24 |
| NO4 | 46 | Male | IV | Adenocarcinoma | TPM3-ROS1 | MET amplification | 12.54 | PR | 11 |
| NO5 | 49 | Female | IV | Adenocarcinoma | CD74-ROS1 | MET amplification | 16.92 | PR | 5 |
| NO6 | 49 | Female | IV | Adenocarcinoma | CD74-ROS1 | KRAS | 9.95 | SD | 4 |
| NO7 | 30 | Female | IV | Adenocarcinoma | CD74-ROS1 | KRAS | 3.23 | PD | 2 |
| NO8 | 50 | Female | IV | Adenocarcinoma | CD74-ROS1 | KRAS | 15.48 | PR | 11 |
| NO9 | 29 | Female | IV | Adenocarcinoma | CD74-ROS1 | KRAS | 1.24 | PR | 33 |

**Table S12**. Detailed clinicopathological characteristics and clinical outcomes of the 9 patients with concomitant driver mutations.

| Patient number | Age | Sex | Clinical stage | Histology | Baseline brain metastasis | Baseline extracranial metastatic site | Best response | PFS (months) | Recurrent extracranial metastases site |
| --- | --- | --- | --- | --- | --- | --- | --- | --- | --- |
| NO1 | 57 | Male | IV | Adenocarcinoma | NO | NO | PR | 14 | Bone |
| NO2 | 66 | Female | IV | Adenocarcinoma | NO | Bone | PR | 12 | Lymph node |
| NO3 | 55 | Female | IV | Adenocarcinoma | NO | NO | PR | 7 | Liver |
| NO4 | 72 | Male | IV | Adenocarcinoma | YES | NO | SD | 4 | Bone |
| NO5 | 50 | Female | IV | Adenocarcinoma | NO | NO | PR | 11 | Liver |
| NO6 | 49 | Female | IV | Adenocarcinoma | YES | Bone | PR | 5 | Peritoneal |
| NO7 | 55 | Female | IV | Adenocarcinoma | YES | Lymph node | PR | 9 | Liver |
| NO8 | 39 | Female | IV | Adenocarcinoma | NO | NO | PR | 4 | Peritoneal |

**Table S13**. Clinicopathological characteristics of the patients with brain progression and non-brain progression on first-line crizotinib therapy

| Clinicopathological characteristics | Intracranial-only progression | | | | Extracranial-only progression | | | | *p*-value |
| --- | --- | --- | --- | --- | --- | --- | --- | --- | --- |
|  | All (n=40) | With baseline brain metastasis (n=16) | Without baseline brain metastasis (n=24) | *p*-value | All (n=64) | With baseline brain metastasis (n=16) | Without baseline brain metastasis (n=48) | *p-*value |  |
| Age-yr (median, range) | 51(33-72) | 52(33-72) | 51(35-68) | 0.753 |  | 52(42-63) | 55(29-78) | 0.786 | 0.793 |
| Sex |  |  |  | 0.76 |  |  |  | 0.587 | 0.612 |
| Male | 16(40) | 7(43.7) | 9(37.5) |  | 23(50) | 7(46.7) | 16(34.8) |  |  |
| Female | 24(60) | 9(56.3) | 16(62.5) |  | 41(50) | 9(53.3) | 32(65.2) |  |  |
| Clinical stage |  |  |  | / |  |  |  | 0.649 | 0.029 |
| III | 0(0) | 0(0) | 0(0) |  | 6(9.3) | 1(6.3) | 5(10.9) |  |  |
| IV | 40(56) | 16(100) | 24(100) |  | 58(90.7) | 15(93.7) | 43(89.1) |  |  |
| ECOG PS score |  |  |  | 0.912 |  |  |  | 0.956 | 0.824 |
| 0-1 | 39(97.5) | 15(93.7) | 24(100) |  | 59(92.1) | 15(93.7) | 44(91.7) |  |  |
| ≥2 | 1(2.5) | 1(6.3) | 0(0) |  | 5(7.9) | 1(6.3) | 4(8.3) |  |  |
| Tumor histology |  |  |  | / |  |  |  | 0.956 | 0.956 |
| Adenocarcinoma | 40(100%) | 16(100) | 24(100) |  | 63(98.5) | 16(100) | 47(98) |  |  |
| Squamous cell carcinoma | 0(0) | 0(0) | 0(0) |  | 1(1.5) | 0(0) | 1(2) |  |  |
| Presence of brain metastasis at baseline |  |  |  | 0.000 |  |  |  | 0.000 | 0.475 |
| Absent | 24(60.0) | 0(0) | 24(100) |  | 48(75) | 0(0) | 48(100) |  |  |
| Present | 16(40.0) | 16(100) | 0(0) |  | 16(25) | 16(100) | 0(0) |  |  |
| Method used for evaluating brain metastasis |  |  |  | 0.596 |  |  |  | 0.577 | 0.964 |
| CT | 13(32.5) | 3(18.7) | 9(37.5) |  | 21(32.8) | 4(25) | 17(35.4) |  |  |
| MRI | 27(67.5) | 13(81.3) | 15(62.5) |  | 43(67.2) | 12(75) | 31(64.6) |  |  |
| Local therapy received for management of brain metastasis |  |  |  | 0.412 |  |  |  | 0.505 | 0.32 |
| none | 34(85.0) | 10(62.5) | 24(100) |  | 63(98.4) | 12(75) | 48(100) |  |  |
| WBRT | 4(10.0) | 4(25.0) | 0(0) |  | 3(2.5) | 3(13.8) | 0(0) |  |  |
| SBRT | 2(5.0) | 2(12.5) | 0(0) |  | 1(1.5) | 1(6.2) | 0(0) |  |  |
